# Oral mucositis & oral health related quality of life in women undergoing chemotherapy for breast cancer in Karachi, Pakistan: A multicenter hospital based cross-sectional study

**DOI:** 10.1371/journal.pone.0295456

**Published:** 2024-04-16

**Authors:** Asad Allana, Uzma Shamsi, Yasmin Rashid, Farhan Raza Khan, Shafquat Rozi

**Affiliations:** 1 Department of Community Health Sciences, Aga Khan University Karachi, Karachi, Pakistan; 2 Department of Oncology, Aga Khan University Karachi, Karachi, Pakistan; 3 Department of Surgery, Section of Dental Surgery, Aga Khan University Karachi, Karachi, Pakistan; University of Catania: Universita degli Studi di Catania, ITALY

## Abstract

**Background:**

Oral mucositis is an inflammatory condition of oral cavity which is a common and serious side effect of cancer treatment. Severe oral mucositis compromises basic functions like eating and swallowing causing malnutrition also affecting overall patient’s oral health related quality of life. The aim of the study was to find the frequency of oral mucositis in patients with breast cancer during their chemotherapy, the factors associated with oral mucositis & the overall patient’s oral health related quality of life.

**Methods:**

A cross-sectional study was conducted and a total of 160 women diagnosed with breast cancer, receiving chemotherapy and who had undergone at least one cycle of chemotherapy were recruited from two hospital settings. In-person interviews were done, patients were asked questions about their sociodemographic history, personal habits, oral history and oral findings, breast cancer stage, chemotherapy history and Oral Health Related Quality of Life. Their oral examination was done at the end of the interview to assess presence or absence of oral mucositis, using World Health Organization oral mucositis tool. Oral Health Related Quality of Life was assessed using Oral Health Impact Profile-14 questionnaire.

**Results:**

Our results showed that out of 160 patients 88 (55%) of the breast cancer cases developed oral mucositis during chemotherapy. The mean Oral Health Impact Profile -14 scores in patients with oral mucositis was high 18.36±0.96 showing poor Oral Health Related Quality of Life. Occasional frequency of brushing was significantly associated with oral mucositis (Prevalence ratio:2.26, 95%_CI 1.06–4.84) compared to those patients who brushed once and twice daily. Low level of education showed negative association with oral mucositis (Prevalence ratio:0.52, 95%_CI 0.31–0.88).

**Conclusion:**

Our study showed significant positive association of occasional brushing with OM and protective association of low level of education with the development of OM. Emphasis should be given to oral hygiene instructions and dental education to cancer patients in oncology clinics with the prescription of mouth washes, gels and toothpaste to patients to decrease OM during chemotherapy.

## Background

Women with breast cancer (BC) who undergo chemotherapy are prone to many oral manifestations including oral mucositis (OM). Chemotherapeutic drugs affect the mouth’s tissue barrier, causing severe OM and gingivitis, bacterial infections, cellulitis, viral mucosal eruptions, and periodontal disease. A study conducted by Saito *et al* showed that approximately 40 percent of patients receiving chemotherapy have been reported to experience these adverse reactions in the oral cavity, with approximately half of patients experiencing serious OM requiring improvement, pause, or discontinuation of therapy [1].Another study conducted by Çakmak, S.*et al* showed that half of the patients i.e. around 51.7% of the patients undergoing cancer chemotherapy developed OM [2].There are many factors leading to development of OM in patients with BC which includes frequency of oral hygiene measure used and the type of chemotherapy as the most important factor. Other important factors associated with BC include age, education level, xerostomia, neutrophil count & white blood cell count at the base line [3].

OM is an inflammatory process of oral cavity caused by chemotherapy. OM is characterized by generalized erythema, pseudo-membranous degeneration, frank ulceration, and hemorrhage. OM is usually observed within 3–5 days after the initiation of chemotherapy and reaches its peak intensity at 7–14 days, affecting on overall oral health related quality of life (OHRQOL) score [4]. Any region of the moveable mucosa can be affected, although the soft palate, ventral and lateral margins of the tongue, and buccal mucosa are the most frequent areas. [5]. OM impairs the function and integrity of the oral cavity, affects the Quality of Life (QoL), and raises morbidity by causing anorexia, dehydration, and malnutrition. Severe OM compromises basic functions like eating and swallowing, as well as social engagement and emotional well-being. OM has a severe effect on patients’ QOL, affecting a variety of daily and psychosocial functions [6].

Chemotherapy induced OM also affects the nutritional status of patients. Failure to evaluate and monitor the ongoing treatment status of soft and hard oral tissues and to take adequate precautions can have profound effects on therapy [7]. The most common chemotherapeutic drugs used to treat early BC include Anthracyclines. This class of drugs includes doxorubicin (Adriamycin) and epirubicin (Ellence) & Taxanes which include docetaxel (Taxotere) and paclitaxel (Taxol). These drugs are often used with other chemo agents like carboplatin, cyclophosphamide (Cytoxan), and fluorouracil (5-FU) [8].

Quality of life comprises an individual’s perceived level of physical, psychological, social, and existential functioning [9]. OHRQol has been used to assess quality of oral health during BC treatment. Assessing OHRQoL as a supplement to normative measures allows for a better understanding of the overall impact of oral disorders on the lives of breast cancer patients. OHRQoL is stated as a multidimensional construct that reflects people’s comfort with their oral health as well as their comfort when eating, sleeping, and interacting with others. [10]

Chemotherapy has reduced the mortality rate of cancer patients; however, OM is an important complication of chemotherapy among women during their chemotherapy cycles and reduce their OHRQOL hindering cancer treatment & care. Chemotherapy induced OM is a preventable condition through a combination of giving regular oral hygiene instructions, counselling of patients, performing regular dental checkups & giving necessary referrals for oral treatments to the patients through which we can effectively prevent OM. Pakistani women undergoing chemotherapy for BC treatment are usually unaware of the side effects of chemotherapy like OM therefore through this study it is important assess frequency of OM caused by chemotherapy and the factors associated with OM among women with BC receiving chemotherapy.

The aim of this study was to determine the frequency of OM and the factors associated with OM among patients receiving chemotherapy for BC with or without the diagnosis of OM after at least one cycle of chemotherapy and to assess OHRQoL using OHIP 14 score in women undergoing chemotherapy for BC with or without OM after at least 1 cycle of chemotherapy at AKUH & JPMC in Karachi in 2021.

## Methods

**Fig 1:**
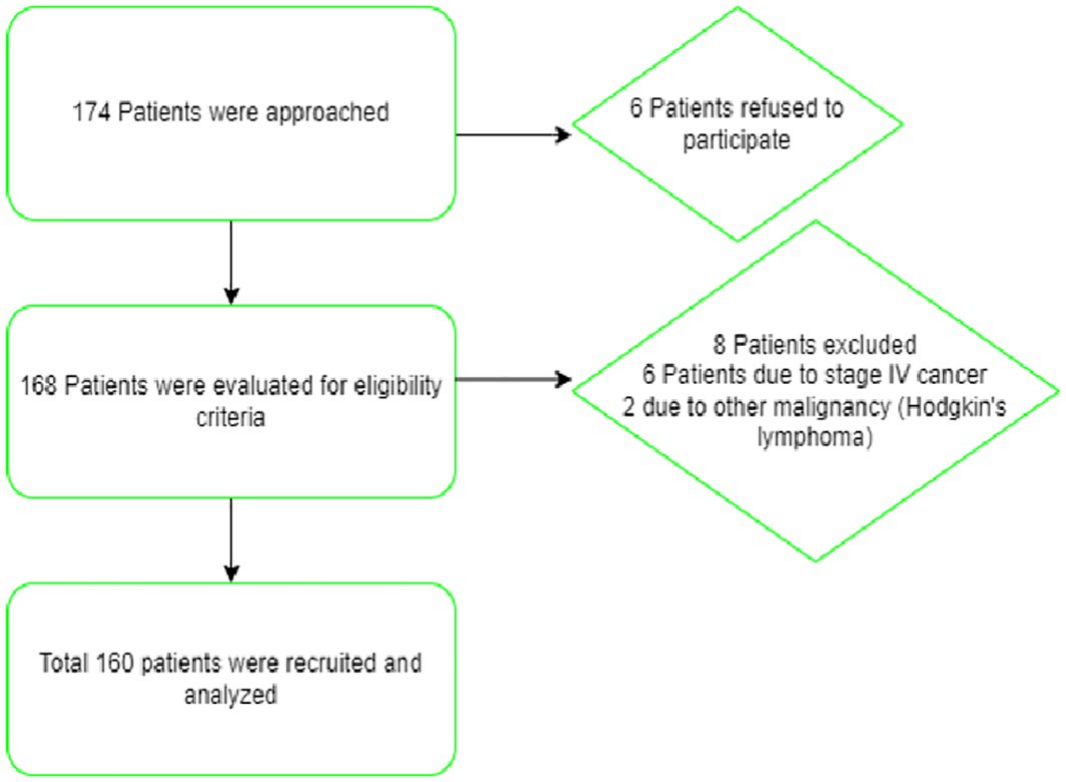
Patient flow chart. Flow chart of the patients.

A cross-sectional study was conducted for 3 months from July 20^th^ to October 20^th^, 2021, in Department of oncology Aga Khan University Hospital (AKUH) & department of oncology Jinnah Postgraduate Medical Center (JPMC) to assess OM in patients with BC receiving chemotherapy. A total of 174 patients with BC were approached for the study from which 6 patients refused to participate in the study. 168 patients were evaluated for the eligibility criteria. 8 patients were excluded, 6 due to stage 4 cancer and 2 due to other malignancy which was our exclusion criteria. Final of 160 patients with BC were recruited and analyzed for OM & OHRQOL as shown in Fig 1, patient flow chart.

Patients were recruited from two hospital settings AKUH and JPMC. Permission was taken from the oncology department of the two hospitals. Women meeting the eligibility criteria were approached for the informed consent and recruited after consent was obtained. After informed consent, patients were interviewed without intervening the pace of clinics. Data collection included in person interviews and oral examination, both were done by principal investigator and research associate who was a dentist in JPMC. In the interview, patients were asked about their socio demographics history, types of oral related hygiene measures and frequency of oral hygiene measures. Additional information about the use of mouth washes and gels was also asked/recorded from medical files. In AKUH, patients were already informed regarding the oral complications such as OM during chemotherapy and were prescribed with mouthwashes, oral drops and gels by doctors, dentist and dental hygienist. However, in JPMC oral hygiene care was not provided to the patients. So, oral counselling was provided to patients who were undergoing chemotherapy. They were educated about brushing technique and oral hygiene. Mouthwashes and gels were prescribed after the interview and oral examination.

Inclusion criteria included all women above 18 years with confirmed case of BC with ongoing chemotherapy who had consented to participate, patients with Stages I-III breast cancer planned to receive neoadjuvant or adjuvant systemic chemotherapy, women who had undergone at least 1 cycle of chemotherapy Exclusion criteria for the study included those women with past history of receiving chemotherapy for any other malignancy, women with any type of other malignancy, women with stage IV and recurrent BC.

Non-probability purposive sampling strategy was used for this study. Sample size was calculated using open epi version 3, For the frequency of OM Significance level of 5%, bound of error 8%, frequency of 40% & 10% non-response rate was taken. Sample size calculated was 160 patients [10].

### Tools

**Fig 2:**
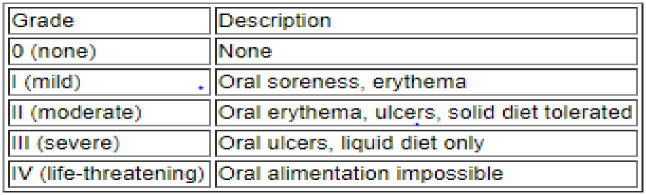
OM tool. The World Health Organization (WHO) grading scale for measuring OM.

The World Health Organization (WHO) grading scale for OM was used for this study, it is based upon clinical features and functionality. The scale measures components that are anatomical, symptomatic and functional related to OM [11]. Patients were examined for the presence or absence of OM using the WHO tool as shown in Fig 2, OM tool.

**Fig 3:**
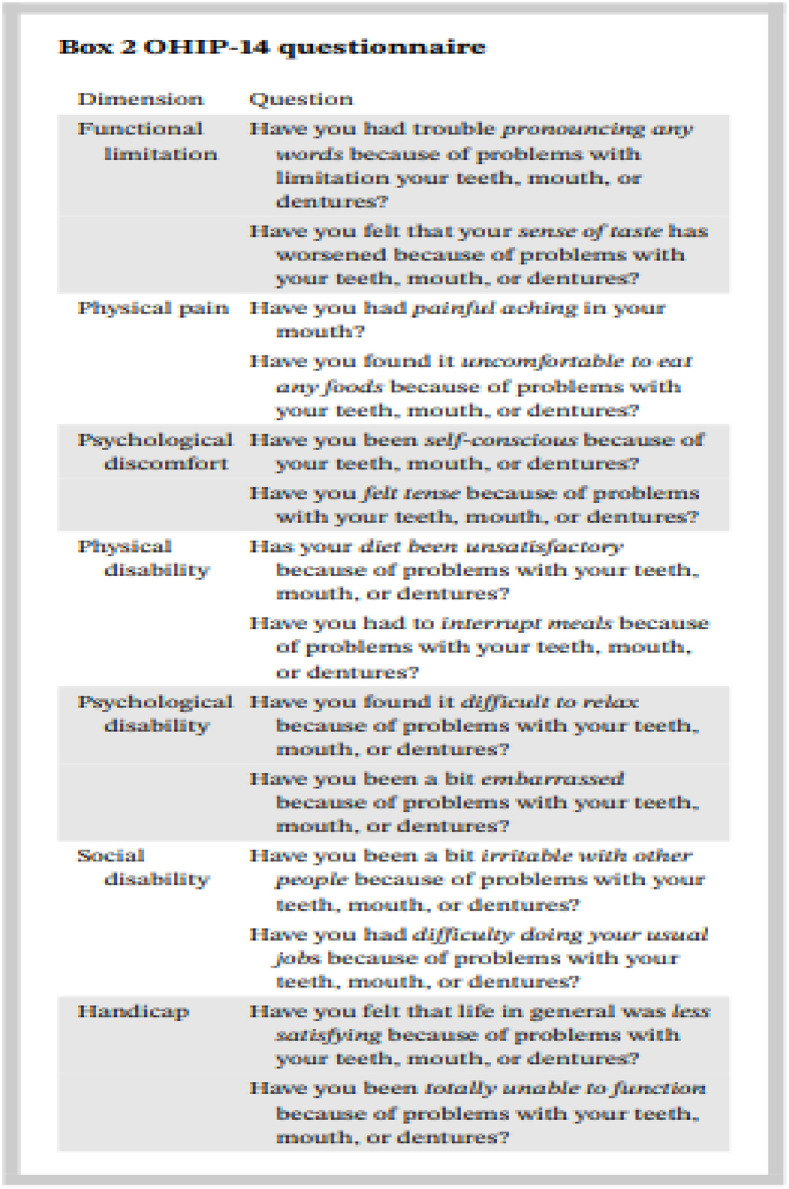
OHIP-14 tool. Oral Health Impact Profile to measure OHRQoL.

Another important variable was OHRQoL. Oral health impact profile -14 (OHIP-14) tool was used to measure OHRQ0L.OHIP-14 is a shorter version of OHIP-49, defined by Slade and Spencer, but maintains the original conceptual dimensions found in OHIP-49. OHIP-14 tool consists of 7 dimensions with two questions each. The seven dimensions are: “Functional limitation”, “physical pain”, “psychological discomfort”, “physical disability” “psychological disability”, “social disability” & “impairment”. A 5-point scale scoring system is followed for OHIP-14, which is coded as 0 = never, 1 = hardly ever, 2 = occasionally, 3 = fairly often, and 4 = very often as shown in Fig 3 OHIP-14 tool. [10]. The score of ≥ 11 shows overall poor OHRQoL and the score of <9.33 was considered a good score for overall OHRQoL [12]. OHIP-14 was translated in Urdu and translated questionnaire underwent content validation by a panel of experts, which revealed a score of 0.95 in relevance and 0.98 in clarity.

### Variables

Our main exposure variable was frequency of oral hygiene measures used. Other variables included were education level, socio-economic status, age, menopausal status, frequency of oral hygiene measures used, gingival status, Decayed Missing Filled Teeth [DMFT], smokeless tobacco consumption, white blood cell count &neutropenia categorical, type of chemotherapy, cycles of chemotherapy.

### Statistical analysis

Data analysis was performed on Stata version 16. We summarized the descriptive statistics (mean and standard deviation) for continuous variables e.g. age, OHIP-14 score, & cycles of chemotherapy. For categorical variables such as OM, education level, employment status, menopausal status, stage of cancer, type of chemotherapy, neutropenia, frequency and proportions were reported. Cox-proportional hazard analysis was done to calculate the PR (Prevalence Ratio) and the 95% CI (Confidence Interval) for each exposure associated with OM e.g., oral care provided by doctors/nurses, frequency of brushing, level of education, age, OHRQoL etc.

### Ethical consideration

Following the approval from DRC (Departmental Review Committee), ethical approval was taken from the Ethical review committee (ERC) of the two hospitals i.e., AKUH and JPMC. The study began after clearance was received from both hospitals. Training sessions for data collector was held to regulate the study in an ethical manner. Data was obtained after participants provided written informed consent. To ensure the confidentiality of the patient’s personal information, each participant was assigned a unique identification number. Participants were assured that they had the full right to withdraw participation in this research at any time before or during the study without incurring any loss or harm in terms of service provision.

## Results

A total of 160 patients with BC were recruited in the study to assess frequency of OM, all the factors associated with OM & overall OHIP-14 score among patients with BC. The main exposure variable was frequency of oral hygiene measures used by patients with BC receiving chemotherapy. OM was present in 88(55%) patients and 72(45%) patients did not develop OM. The mean score & standard deviation of overall OHIP-14 score among patients who developed OM was higher (18.36±0.96) compared to (12.12±0.76) patients who did not develop OM.

Table 1 shows sociodemographic factors, type of oral hygiene measures, oral findings & family history of BC women.

**Table 1:** Sociodemographic characteristics, personal habits, oral related hygiene measures, oral findings, breast cancer stage & chemotherapy history of 160 patients with BC in a multicenter cross-sectional study in Karachi Pakistan.

SNO	Variables	OM Present (88) n %	OM Absent (72) n %	*p-value*
**1**	Age in years*			
	(Mean±SD)	47.87±1.18	47.36±1.25	0.84
**2**	**Socio-economic status**			0.68
	Low	53 (60.2)	42 (58.3)	
	Middle	19 (21.6)	21 (29.2)	
	High	16 (18.2)	9 (12.5)	
**3**	**Marital status**			0.76
	Single	8 (9.1)	4 (5.5)	
	Married	66 (75.0)	53 (73.6)	
	Separated /widowed	14 (15.9)	15 (20.8)	
**4**	**Menopausal status**			0.84
	Pre-menopausal	42 (47.7)	36 (50.0)	
	Post-menopausal	46 (52.3)	36 (50.0)	
**5**	**Level of education**			0.03
	Schooling /matric	50 (56.8)	59 (81.9)	
	Intermediate	9 (10.2)	8 (11.1)	
	Graduate/post-graduation	29 (32.9)	5 (6.9)	
**6**	**Employment status**			0.40
	Un-employed	78 (88.6)	68 (94.4)	
	Employed	10 (11.6)	4 (5.5)	
	**Personal habits, oral history & oral findings**			
**7**	**Use of smokeless tobacco (Paan, betel nut, areca nuts/gutka)**			
	No	76 (86.3)	63 (87.5)	0.88
	Yes	12 (13.6)	9 (12.5)	
**8**	**Oral hygiene measure used**			
	Miswak /dentonic	10 (11.3)	15 (20.8)	0.25
	Brush	78 (88.6)	57 (79.2)	
**9**	**Frequency of oral hygiene measure used**			0.09
	Occasionally	28 (31.8)	13 (18.1)	
	Once daily	50 (56.8)	38 (52.7)	
	Twice daily	10 (11.3)	21 (29.2)	
**10**	**Oral care provided by doctors/nurses**			0.08
	No	25 (28.4)	8 (11.1)	
	Yes	63 (71.6)	64 (88.9)	
**11**	**DMFT (Decayed Missing Filled Teeth)**			0.98
	Low (0.0–2.6)	17 (19.3)	13 (18.1)	
	Moderate (2.7–4.4)	4 (4.5)	3 (4.2)	
	High (4.5 & >)	67 (76.1)	56 (77.8)	
**12**	**Gingival status**			0.11
	No inflammation	36 (40.9)	47 (65.3)	
	Mild inflammation	41 (46.6)	21 (29.2)	
	moderate to severe inflammation	11 (12.5)	4 (5.5)	
**13**	OHIP-14 score*			
	(Mean±SD)	18.36±0.96	12.12±0.76	0.002
	**Breast cancer stage & chemotherapy history**			
**14**	**Stage of cancer**			0.80
	Stage 1	3 (3.4)	4 (5.5)	
	Stage 2	40 (45.4)	28 (38.9)	
	Stage 3	45 (51.5)	40 (55.5)	
**15**	Cycles of chemotherapy*			0.37
	(Mean±SD)	8.78±0.58	7.58±0.68	
**16**	**Type of chemotherapy**			
	• Taxanes only	21 (23.8)	20 (27.7)	0.15
	• AC only	11 (12.5)	20 (27.7)	
	• AC+Taxanes	56 (63.6)	32 (44.4)	
**17**	**White Blood Cell count**			0.82
	Low (<4.5x10^9^/L)	16 (18.2)	15 (20.8)	
	Normal (4.5x10^9^/L to 11x10^9^/L)	67 (76.1)	55 (76.4)	
	High (> 11x10^9^/L)	5 (5.7)	2 (2.8)	
**18**	**Neutrophil count**			0.42
	Low (<50%)	9 (10.2)	5 (6.9)	
	Normal (50–75%)	70 (79.5)	65 (90.3)	
	High (>75%)	9 (10.2)	2 (2.8)	
**19**	**Platelet count**			0.92
	Low(<140x10^9^/L)	4 (4.5)	2 (2.8)	
	Normal(140x10^9^/L-400x10^9^/L)	68 (77.3)	56 (77.8)	
	High(>400x10^9^/L)	16 (18.2)	14 (19.4)	

Abbreviations: OM, Oral Mucositis; DMFT, Decayed Missing Filled Teeth, OHIP-14, Oral Health Impact Profile-14.

Mean age of patients with OM was 47.87 ± 1.18 years, while that of patients without OM was 47.36± 1.25 years. For socioeconomic status, 60.2% patients were from low socio-economic and 18.2% were of high socio-economic status who developed OM. Around 47.7% of the pre-menopausal women and 52.3% of the post-menopausal women developed OM during chemotherapy treatment. In patients who developed OM 56.8% had done primary schooling or matriculation & 32.9% were graduates or postgraduates. Around 13.6% of the patients who consumed smokeless tobacco (pan/betel nuts/areca nuts/gutka) developed OM while 12.5% did not develop OM. Nearly 88.6% patients who developed OM reported brushing their teeth while 11.3% used other oral hygiene measures such as miswak/powder, 79.12% patients reported brushing their teeth while 20.83% used other oral hygiene measures such as miswak/powder.

Nearly 31.8% patients with OM, reported using oral hygiene measures such as brushing/miswak/powder occasionally while 56.8% used oral hygiene measures once daily. On the other hand, 18% of the patients with no OM, reported using oral hygiene measures such as brushing/miswak/powder occasionally while 52.7% used oral hygiene measures once daily.

Oral care provided to 71.6% of the patients during chemotherapy like oral hygiene instruction, mouthwashes, gels developed OM while in 88.9% of the patients in whom oral care was provided did not develop OM. High DMFT score was found in 76.1% of the patients who develop OM. While 77.8% patients with high DMFT score did not develop OM. Around 12.5% of the patients who developed OM had moderate/severe gingival inflammation while 5.5% who did not develop OM had moderate/severe gingival inflammation.

Around 51.5% of the patients with OM had stage 3 breast cancer while 45.5% had stage 2 cancer. The mean number of chemotherapy cycles in patients with OM was (8.78±0.58 cycles) compared to (7.58±0.68 cycles) in patients without OM. Around 63.6% who received AC (Adriamycin/Cyclophosphamide) + taxanes and 23.8% of the patients who received taxanes only, developed OM. Nearly half of the patients (44.4%) patients who received AC + taxanes and 27.7% patients who received taxanes only did not develop any OM. Around 18.18% of the patients with low WBC (White Blood Cell) count developed OM while 20.8% of the patients with low WBC count did not develop OM, while 10.2% of the patients with low neutrophil count developed OM compared to 6.9% of the patients with low neutrophil count did not develop OM. Around 4.5% of the patients with low platelet count developed OM while 2.7% of the patients with low platelet count did not develop OM.

Our final model (Table 2) showed that the prevalence of OM in patients whom the frequency of oral hygiene measure was practiced occasionally increased by 2.26 (95% CI 1.06–4.84) compared to patients who use oral hygiene measures twice daily when controlling for level of education & oral care provided by doctors/nurses & the prevalence of OM in patients whom the frequency of oral hygiene measure was practiced once daily increased by 1.59 (95% CI 0.37–1.79) compared to patients who use oral hygiene measures twice daily when controlling for level of education & oral care provided by doctors/nurses.

**Table 2:** Multivariable analysis of OM and factors associated with OM among 160patients with BC in a multicenter cross-sectional study in Karachi Pakistan.

Variable	p-value	PR (95% CI)
Level of education*		
Schooling/matric	0.011	0.52 (0.31,0.85)
Intermediate	0.429	0.73 (0.33,1.59)
Graduate/Postgraduate		(reference)
Frequency of oral hygiene measure*used		
Occasionally	0.014	2.55 (1.21,5.37)
Once daily	0.075	1.78 (0.93, 3.76)
Twice daily		(reference)
**Oral care provided by doctors/nurses**		
No		1.22 (0.74,2.02)
Yes	0.427	(reference)

Abbreviations: PR, Prevalence Ratio; CI, Confidence interval

The prevalence of OM among those patients who had either primary schooling or matriculation certificate is 0.52 (95% CI 0.31 0.81) compared to patients who were graduates/post graduates when controlled for frequency of oral hygiene measure used & oral care provided by doctors/nurses & the prevalence of OM among those patients who had done intermediate is 0.84 (95% CI 0.31 0.81) compared to patients who were graduates/post graduates when controlled for frequency of oral hygiene measure used & oral care provided by doctors/nurses.

Oral care provided by doctors/nurses was statistically insignificant, but was kept in the final model due to being an important variable in development of OM. Our results showed that the prevalence of OM in patients in whom oral care was not provided by doctors/nurses increased by 1.11 (95% CI 0.66 1.87) compared to patients in whom oral care was provided by doctors/nurses when controlling for level of education & frequency of oral hygiene measure used.

## Discussion

Our results showed that 55% (88) of the BC women developed OM during chemotherapy. This result is comparable with similar study conducted by Çakmak *et al* which reported that 51.7% of the patient’s undergoing chemotherapy developed OM [2]. Our results showed that the mean overall OHIP-14 score was higher (18.36±0.96) in the patients with OM compared to patients without OM (12.12±0.7). The score was high for both categories, but was greater for OM group. A study conducted by Priya Sahni *et al* showed similar results, reporting that in patients with oral alterations after chemotherapy the overall OHIP-14 score was higher (12.4 ±7.4) compared to the OHIP-14 score before chemotherapy (4.0± 7.7) [10].

The mean age of the patients was similar 47.87±1.18 for the OM group and 47.36±1.25 for the patients without OM. As most of the women were of middle age our results were inconsistent with the previous literature as suggested by Lorini L *et al* that the risk developing OM is high in young age due to a faster rate of cell regeneration and also in old age due to a slower pace of healing process [13]. Our results showed that 60.2% of the patients who developed OM were of low socio-economic status and 58.3% of the patients from low socio-economic status did not develop OM. A study conducted by Howard JR *et al* showed that low socio-economic status is the key factor associated with oral health in cancer patient. Low socioeconomic level is the most important factor related with poor oral health in cancer patients. Low-income patients cannot afford dental treatment or receive sufficient oral care information from their oncologists, exacerbating the situation [14].

The frequency of patients consuming smokeless tobacco was similar for both groups. Our results showed that 13.6% of the patients who consumed smokeless tobacco developed OM and 12.5% of the patients who consumed smokeless did not developed OM. A study conducted by Sujatha S Reddy *et al* showed that increase in use of smokeless tobacco causes increase in risk of oral disorders such as OM & OSF (Oral Sub Mucous Fibrosis) [15]. The Final DMFT score was similar and high for both groups.76.1% of the patients had high DMFT score in OM group compared to 77.8% patients without OM. A study conducted by Ines Willershausen *et al* showed that Patients undergoing treatment for cancer are more likely to have a high DMFT score. Cancer patients exhibited significantly more apical lesions without root canal fillings and lost teeth than healthy controls. Tooth decay and gum disease are the major causative factor of OM [16].

Our results showed that 12.5% of the patients with moderate to severe gingival inflammation developed OM while 5.5% patients with moderate to severe gingival inflammation did not developed OM. A study conducted by Coracin *et al* supported these results which showed that poor gingival and periodontal dental health are predictive factors for the occurrence and severity of OM [17]. Our results showed that the mean no of chemotherapy cycles in both groups were similar. The mean no of chemotherapy cycles 8.78±0.58 for the OM group and 7.58±0.68 for the patients who did not developed OM. A study conducted by Burciaga *et al* showed opposite results. The study showed that the risk of developing OM is high within first six cycles of chemotherapy compared to greater than six cycles [18]. Our result showed that 63.6% of the patients receiving AC +taxanes both develop OM and 44.4% receiving both doses did not develop OM. A study conducted by Nishimura *et al* showed that risk of developing OM increased when the patient received AC (doxorubicin hydrochloride and cyclophosphamide) treatment regimen [19].

Our results showed that 10.2% of the patients with low neutrophil count developed OM compared to 6.9% of the patients with low neutrophil count did not developed OM. The results were consistent with the previous literature, as suggested by Sampson *et al* showed that mucositis is a common consequence in neutropenic individuals receiving immunosuppressive treatment, leading to mouth ulcers and periodontal infections [20]. However, all these variables were statistically insignificant.

Our results showed that prevalence of OM in patients in whom oral care was not provided by doctors/nurses increased by 1.22 (95% CI 0.74–2.02) compared to patients in whom oral care was provided by doctors/nurses when controlled for level of education & frequency of oral hygiene measure used. This result was statistically insignificant and was inconsistent with the previous literature a randomized control trial conducted by Saito H *et al* which showed that that starting prophylactic professional oral health care (POHC) before chemotherapy significantly reduced oral deterioration and the incidence of oral mucositis. These results support the efficacy of regular prophylactic POHC in cancer patients. [1].

Our results showed that prevalence of OM in patients whom oral hygiene measures were practiced occasionally increased by 2.55 (95% CI 1.21–5.37) compared to patients who practiced oral hygiene measures once & twice daily when controlled for level of education & oral care provided by doctors/nurses. These results were consistent with the previous as a study conducted by. Murshid *et al* showed that 38.2% patients who were brushed their teeth occasionally within the third week of receiving chemotherapy showed the presence of OM compared to 3.8% patients who were brushing twice or more per day with a significance of (*p* = 0.002) [21].

Our results showed that the prevalence of OM among those patients who had done either primary schooling or matriculation certificate is 0.52 times (95% CI 0.31 0.85) compared to patients who had done intermediate or were graduates/postgraduates when controlled for frequency of oral hygiene measure used & oral care provided by doctors/nurses. These results were quite opposite with the previous literature as our results showed protective effect of low education with OM development this result could be possible because low educated people are less confident, they listen and follow the instructions more of doctors and nurses compared to people who are highly educated. Previous studies showed that more the person is educated the risk of developing OM is less. A study conducted by Araújo *et al* showed that patients with a higher level of education cope better with cancer diagnoses and are more likely to adhere to medical and nursing instructions and prescriptions, such as for oral cleanliness in those with OM [22]. Another study conducted by Abdelaziz *et al*. also showed that there was highly statistically significant association between patients practice of oral care and educational level with more the person being educated, less the chances of developing OM [23].

The strength of this study is that it did not only measure the frequency of OM and all the factors associated with OM in detail but also the overall OHIP-14 score in the patients with BC. Another strength of this study is that it was a multicenter study conducted in public and private hospital setting representing patients from all socio-economic status increasing the generalizability of our results. Counselling of patients was also done, oral hygiene instructions were given, patients were educated about brushing techniques, mouthwashes and gels were also prescribed to the patients in government hospital.

The major limitation of this study was that it was a cross-sectional study conducted with inherent biases such as recall bias and non-response bias. Another limitation of cross-sectional study design was that temporality was not established between the exposure (frequency of oral hygiene measure used) and outcome (OM). A longitudinal follow-up study would have given better results if we had followed patients with BC for a period of time to measure the effects of type of chemotherapy and number of cycles more accurately on development of OM. If a longer longitudinal follow-up study was conducted and we had done oral examination before and after at least one cycle of chemotherapy, we would have gotten better idea that OM developed due to chemotherapy or there were other factors which lead to OM. Another limitation of our study was that we used non-probability purposive sampling and did not stratify our patients according to government and private hospital setup as most of our patients were from government hospital, based on difference in OM in the 2 hospitals, weighted sample size should have been done and when not done, it may have resulted in overestimation of the OM frequency.

Based on our results with 55% of the patients having OM, the study results emphasize on the multidisciplinary approach in this regard. The role of dentist fits here as half of all chemotherapy patients experienced OM, taste changes and xerostomia indicating that the patient should be consulted by a dentist before beginning the treatment. Patient education by a dentist is necessary to improve OHRQoL. Nurses working in oncology settings also have a critical role in assisting the patients in managing their impaired oral function. They should be providing psychological support, as well as dietary interventions for patients who have difficulties swallowing and supportive care measures for relief of dry mouth and distorted taste. Patients should be informed about OM before the chemotherapy and should be given oral hygiene instructions in which they should be educated about brushing techniques, flossing & use of mouth rinses. Like private hospitals, government hospitals should also conduct regular dental checkups after every chemotherapy cycle and provide mouthwashes and gels to treat OM.

Future direction of the management of OM should focus on evidence-based rehabilitation and pre- and post-chemotherapy monitoring. Focus group discussions and awareness of OM to patients with breast cancer using different videos and pamphlets should be considered.

In summary, the management of cancer-related mucositis is swiftly developing. Because the causes of OM are complex and multifaceted, the prevention strategy must be intended to ensure maximum OM control by manipulating multiple cascade pathways. A variety of approaches is likely to be the future standard of care. At the moment, Oro-dental care, regular mouth rinses, the use of growth agents, and topical treatment may be the answer. A longitudinal, multicentre follow-up studies are also required so temporality is established between chemotherapy and OM for prevention and better management of OM.

## Conclusion

Our study showed significant association between occasional frequency of brushing and development of OM in patients with BC as high OM prevalence was found in the patients who did not take care of their oral hygiene and brushed occasionally. Our study also showed negative association between low level of education and development of OM which was quite an unusual finding. Emphasis should be given to oral hygiene instructions and dental education to cancer patients in oncology clinics. Mouth washes, gels & toothpaste should be provided to patients with OM for early eradication. Nurses should also play an important role in providing psychological support and maintaining nutrition of the patient.

## Supporting information

S1 Data (XLSX)

S2 Data (XLSX)

## Abbreviations

AC: Adriamycin/cyclophosphamide
AKUH: Aga Khan University Hospital
BC: Breast Cancer
CI: Confidence Interval
DMFT: Decayed Missing Filled Teeth
DRC: Departmental Review Committee
ER: Estrogen Receptor
ERC: Ethical Review Committee
JPMC: Jinnah Postgraduate Medical Center
OHIP-14: Oral Health Impact Profile -14
OHRQOL: Oral Health Related Quality of Life
OM: Oral Mucositis
OSF: Oral Submucous Fibrosis
PI: Principal Investigator
PR: Prevalence Ratio
QOL: Quality of Life
TC: Taxotere/cyclophosphamide
WHO: World Health Organization

## References

[1] Saito H, Watanabe Y, Sato K, Ikawa H, Yoshida Y, Katakura A, et al. Effects of professional oral health care on reducing the risk of chemotherapy-induced oral mucositis. Supportive Care in Cancer. 2014 Nov;22:2935–40. doi: 10.1007/s00520-014-2282-4 24854326 PMC4183888

[2] Çakmak S, Nural N. Incidence of and risk factors for development of oral mucositis in outpatients undergoing cancer chemotherapy. International Journal of Nursing Practice. 2019 Feb;25(1):e12710 doi: 10.1111/ijn.12710 30461128

[3] McCarthy GM, Awde JD, Ghandi H, Vincent M, Kocha WI. Risk factors associated with mucositis in cancer patients receiving 5-fluorouracil. Oral Oncol. 1998 Nov;34(6):484–90. doi: 10.1016/s1368-8375(98)00068-2 . 9930359

[4] Barkokebas A, Silva IH, de Andrade SC, Carvalho AA, Gueiros LA, Paiva SM, et al. Impact of oral mucositis on oral-health-related quality of life of patients diagnosed with cancer. Journal of oral pathology & medicine. 2015 Oct;44(9):746–51 doi: 10.1111/jop.12282 25345344

[5] Shankar A., Roy S., Bhandari M., Rath G. K., Biswas A. S., Kanodia R., et al. (2017). Current Trends in Management of Oral Mucositis in Cancer Treatment. *Asian Pacific journal of cancer prevention*: *APJCP*, 18(8), 2019–2026. doi: 10.22034/APJCP.2017.18.8.2019 28843216 PMC5697454

[6] Al Ibraheemi AA, Shamoun S. Incidence and risk factors of oral mucositis in patients with breast cancer who receiving chemotherapy in Al-Bashir hospital. International journal of hematology-oncology and stem cell research. 2016 Oct 10;10(4):217 27928476 PMC5139941

[7] Morais MO, Martins AF, de Jesus AP, de Sousa Neto SS, da Costa AW, Pereira CH, et al. A prospective study on oral adverse effects in head and neck cancer patients submitted to a preventive oral care protocol. Supportive Care in Cancer. 2020 Sep;28:4263–73. doi: 10.1007/s00520-019-05283-1 31900618

[8] Mandapati A, Lukong KE. Triple negative breast cancer: Approved treatment options and their mechanisms of action. Journal of Cancer Research and Clinical Oncology. 2023 Jul;149(7):3701–19 doi: 10.1007/s00432-022-04189-6 35976445 PMC10314854

[9] Phoosuwan N, Lundberg PC. Life satisfaction, body image and associated factors among women with breast cancer after mastectomy. Psycho-Oncology. 2023 Apr;32(4):610–8. doi: 10.1002/pon.6106 36670514

[10] Sahni P, Punyani SR, Jain S, Nayak KC, Charan A, Karwasra K. Oral alterations and oral health-related quality of life assessment in patients undergoing chemotherapy at a tertiary care center. Special Care in Dentistry. 2020 Sep;40(5):450–6. doi: 10.1111/scd.12499 32710806

[11] Pulito C, Cristaudo A, Porta CL, Zapperi S, Blandino G, Morrone A, et al. Oral mucositis: the hidden side of cancer therapy. Journal of experimental & clinical cancer research. 2020 Dec;39:1–5. doi: 10.1186/s13046-020-01715-7 33028357 PMC7542970

[12] Warsi I, Younus A, Rasheed A, Ahmed J, Mahida H, Hashmi R, et al. Oral health-related quality of life in patients with upper gastrointestinal and hepatic disorders in Pakistan: validation of the Oral Health Impact Profile-14 in the Urdu language. BDJ open. 2018 Apr 27;4(1):1–7. doi: 10.1038/s41405-018-0002-8 30057791 PMC5944343

[13] Lorini L, Perri F, Vecchio S, Belgioia L, Vinches M, Brana I, et al. Confounding factors in the assessment of oral mucositis in head and neck cancer. Supportive Care in Cancer. 2022 Oct;30(10):8455–63. doi: 10.1007/s00520-022-07128-w 35639187 PMC9512735

[14] Howard JR, Ramirez J, Li Y, Gany F. Dental care access for low-income and immigrant cancer patients in New York City. Journal of community health. 2015 Feb;40(1):110–5 doi: 10.1007/s10900-014-9904-0 24984598

[15] Reddy SS, Prashanth R, Y Devi B, Chugh N, Kaur A, Thomas N. Prevalence of oral mucosal lesions among chewing tobacco users: A cross-sectional study. Indian Journal of Dental Research. 2015 Sep 1;26(5):537. doi: 10.4103/0970-9290.172083 26672428

[16] Willershausen I, Schmidtmann I, Azaripour A, Kledtke J, Willershausen B, Hasenburg A. Association between breast cancer chemotherapy, oral health and chronic dental infections: a pilot study. Odontology. 2019 Jul;107(3):401–8. doi: 10.1007/s10266-019-00411-z 30666484

[17] Coracin FL, Santos PS, Gallottini MH, Saboya R, Musqueira PT, Barban A, et al. Oral health as a predictive factor for oral mucositis. Clinics. 2013;68:792 doi: 10.6061/clinics/2013(06)11 23778491 PMC3674268

[18] Carreón-Burciaga RG, Castañeda-Castaneira E, González-González R, Molina-Frechero N, Gaona E, Bologna-Molina R. Severity of oral mucositis in children following chemotherapy and radiotherapy and its implications at a single oncology centre in Durango State, Mexico. International Journal of Pediatrics. 2018 May 10;2018. 10.1155/2018/3252765 PMC5971240 29861749

[19] Nishimura N, Nakano K, Ueda K, Kodaira M, Yamada S, Mishima Y, et al. Prospective evaluation of incidence and severity of oral mucositis induced by conventional chemotherapy in solid tumors and malignant lymphomas. Supportive Care in Cancer. 2012 Sep;20(9):2053–9. doi: 10.1007/s00520-011-1314-6 22116139

[20] Sampson MM, Nanjappa S, Greene JN. Mucositis and oral infections secondary to gram negative rods in patients with prolonged neutropenia. IDCases. 2017 Jan 1;9:101–3. doi: 10.1016/j.idcr.2017.06.014 28736716 PMC5512178

[21] Murshid EZ, Azizalrahman TA, AlJohar AJ. Oral mucositis in leukemic Saudi children following chemotherapy. The Saudi Journal for Dental Research. 2017 Jan 1;8(1–2):79–85.

[22] Araújo SN, Luz MH, Silva GR, Andrade EM, Nunes LC, Moura RO. Cancer patients with oral mucositis: challenges for nursing care. Revista latino-americana de enfermagem. 2015 Feb; 23:267–74. doi: 10.1590/0104-1169.0090.2551 26039297 PMC4459000

[23] Abdelaziz sh. Oral care and its association with socio-demographic characteristics in leukemic patients receiving chemotherapy. The Malaysian Journal Of Nursing (MJN). 2020 oct 1;12(1):98–105.

